# Editorial: Rethinking unsuccessful psychotherapies: when and how do treatments fail?

**DOI:** 10.3389/fpsyg.2024.1514654

**Published:** 2024-11-20

**Authors:** Osmano Oasi, Kenneth L. Critchfield, Andrzej Werbart

**Affiliations:** ^1^Department of Psychology, Catholic University of the Sacred Heart, Milan, Italy; ^2^Ferkauf Graduate School of Psychology, Yeshiva University, New York, NY, United States; ^3^Department of Psychology, Faculty of Social Sciences, Stockholm University, Stockholm, Sweden

**Keywords:** dropout, treatment failure, side-effects, psychotherapeutic relationship, psychopathology

As underscored in our previous Research Topic on the issue (Oasi and Werbart, 2020), treatment failure remains a neglected subject in psychotherapy research, despite fact that much can be learned from failed treatments. It is likely variables pertaining to characteristics of psychotherapists, patients, their relationship together, and the implementation of specific techniques in specific context all relate to unsuccessful treatments, just as they do with successful ones (Castonguay and Beulter, 2005).

The topic is not new. Patient and therapist variables are considered in early psychoanalytic studies such as in Breuer's case of Anna O., and Dora's drop out from treatment with Freud. A few years ago, Goldberg (2012) came back to this Research Topic and proposed a “taxonomy of treatment failures.” Referring to the psychoanalytic concept of *impasse*, he identified patient and therapist contributions to dropout. More recently, the journal *Psychotherapy Research* devoted a special section to premature termination (Swift et al., 2018). However, the needs in this area are greater than the attention paid to date. One systematic review observed that only 57 of 1,430 relevant publications monitored negative effects (Honkalampi et al., 2024). Meanwhile, one recent study estimated adverse events for 1 in 21 patients in psychotherapy RCTs (Klatte et al., 2023) while another study found that 96% of patients in cognitive-behavior, psychodynamic and psychoanalytic therapy reported at least one negative side-effect (Wittmann et al., 2023). These data underscore the need for greater attention to the “dark side” of treatment outcome and effects.

The current Research Topic contains work from researchers in England, Germany, Italy, and the Netherlands. Four of the seven studies focus on work with adult outpatients, while 3 involve the challenges of work with adolescents. Problems treated range from mild-to-moderate depression and anxiety, to conduct disorder in adolescents (Hauschild et al.) and personality disorder in adults (Fiorentino et al.). Methodologies span single-case and small-sample mixed-method inspections (Cirasola et al., Fiorini et al.), to quantitative methods applied with both patient (Hauschild et al., McLeod et al., Verkooyn et al.) and therapist data (Fiorentino et al., Oasi et al.).

Of special interest to us is that treatment failure is defined by these authors in multiple ways. A focus on dropout is reflected in the work of Cirasola et al., who present a mixed-methods analysis of an adolescent treatment in which alliance difficulties persisted despite the therapist's attempts to address them. [Hauschild et al. predicted drop out cases using a cluster analytic approach with a sample of adolescent patients having conduct disorder. Those who dropped out had a profile of notable difficulties with intimacy, empathy, and self-definition. Oasi et al. also focused on dropout, but from the novel perspective of the clinician's experience, finding that a range of countertransference reactions are involved. A cross-cutting theme with dropout cases appears to be a compromised therapy relationship, with multiple contributing factors identified.

Other authors focus on those who complete therapy (Fiorini et al., McLeod et al., Verkooyen et al.). Each of these studies underscores the potential for divergence between symptom change and patient *experience* of therapy. Verkooyan and colleagues provide evidence that negative experiencing in therapy is not associated with symptom change (and is also common). Fiorini et al. provide qualitative data from “non-responder” adolescents who nonetheless valued therapy as a “safe space.” Similarly, McLeod et al. found that perceived helpfulness of a brief counseling intervention did not correlate with symptom change measures among adolescents. And finally, Fiorentino et al. focus on factors that compromise the *depth of experience* in sessions, raising further questions about what constitutes “failure” in therapeutic work. McLeod et al. provide additional commentary that makes clear how definitions of treatment success or failure have important moral, ethical, and social justice implication. These are important to consider alongside findings such as those of a recent meta-synthesis of 24 qualitative studies (Carrington et al., 2024) concluding that treatment nonresponse involves a range of patient and therapist negative experiences.

Our opinion as editors aligns well with those of the special section authors, who in various ways suggest that the language of “treatment failure” represents an umbrella term for a broad array of unwished-for effects of psychotherapy, with contributions from multiple sources. This includes the person of the psychotherapist as noted in the work of Fiorentino et al., who underscore the concept of therapist responsiveness, and Oasi et al. who present hypotheses based on the psychotherapist under pressure (Muran and Eubanks, 2020), with potential connections to narcissism (Oasi et al., 2019).

Future challenges for our field involve the need not only for more research, but for better specification of theory, constructs, and variables underneath the umbrella term of “treatment failure.” These likely include (1) patient variables such as the degree of “epistemic trust” (suggested by Cirasola et al., Fiorini et al., and Hauschild et al.), (2) potential vulnerabilities in psychotherapists themselves (Maroda, 2022; Oasi et al.), or (3) in the way they implement specific techniques (Critchfield et al., 2022; Fiorini et al.). Each of these factors may in turn affect the relational process, for example through therapist ability to empathize with patients and express it in helpful ways. There is also hope of identifying and prospectively monitoring relevant variables from the beginning of treatment (e.g., De Salve et al., 2024) to offset ways in which retrospective methodologies can be affected by bias (Swift and Greenberg, 2012).

Our hope is that this second Research Topic will help advance clinical practice as well as theory and research. If we wish to avoid harm in our efforts to help, we need to understand what we get wrong in treatment as much as what we get right. The authors in this special section make important strides toward the clarity and rigor we need from our science in this area.
